# Retraction Note: LINC00355 induces gastric cancer proliferation and invasion through promoting ubiquitination of P53

**DOI:** 10.1038/s41420-024-01828-4

**Published:** 2024-01-30

**Authors:** Wenjing Zhao, Yan Jin, Peng Wu, Jian Yang, Yuanyuan Chen, Qianlu Yang, Xinying Huo, Juxue Li, Wei De, Jinfei Chen, Fen Yang

**Affiliations:** 1 Department of Oncology, Nanjing First Hospital, Nanjing Medical University, 210006 Nanjing, People’s Republic of China; 2 Department of Biochemistry and Molecular Biology, School of Basic Medical Sciences, Nanjing Medical University, 211166 Nanjing, People’s Republic of China; 3 Cancer Center, Taikang Xianlin Drum Tower Hospital, Nanjing University School of Medicine, 210046 Nanjing, People’s Republic of China; 4 Jiangsu Key Lab of Cancer Biomarkers, Prevention and Treatment, Collaborative Innovation Center for Personalized Cancer Medicine, Nanjing Medical University, 211166 Nanjing, People’s Republic of China

Retraction to: *Cell Death Discovery* 10.1038/s41420-020-00332-9, published online 08 October 2020

The Editors-in-Chief have retracted this article at the corresponding author’s request. After publication, concerns were raised regarding the image overlap within the figures in this article, as well as with two other articles from the same group [1, 2]. Specifically:

Fig. 3D MGC803 LINC00355 siRNA and AGS pcDNA3.1-LINC00355 images appear to overlap with Fig. 4D BGC823 PLZF and SGC7901 Vector, respectively, in the authors’ earlier article [1].

Fig. 3C BGC823 LINC00355 siRNA and AGS pcDNA3.1 appear highly similar.

Fig. 6A Myc-UBE3C Input:Myc blot appears highly similar to Fig. 6C Myc-RAD16 Input:Flag lanes 2 and 3.

Six panels in Fig. 3E appear highly similar to those in Fig. 8E in [2].

The authors have stated that the images in this article were misused.

Further checks by the Publisher have found that the study used MGC803, BGC823 and SGC7901 cell lines, which are contaminated (or partially contaminated) with HeLa cells, and are therefore not a suitable model for gastric cancer; and the ethics approval numbers for both the animal and human studies are missing from this article.

The Editors-in-Chief therefore no longer have confidence in the presented data.

Fen Yang has stated on behalf of all authors that they agree to this retraction.
